# Atypical femur fracture associated with common anti-osteoporosis drugs in FDA adverse event reporting system

**DOI:** 10.1038/s41598-023-37944-x

**Published:** 2023-07-05

**Authors:** Yao Xiao, Yiqian Chen, Yan Huang, Yuan Xiao

**Affiliations:** 1 Department of Endocrinology, Endocrinology Research Center, Xiangya Hospital of Central South University, Changsha, 410008 Hunan China; 2 Department of Gastroenterology, Xiangya Hospital of Central South University, Changsha, 410008 Hunan China; 3 National Clinical Research Center for Geriatric Disorders, Xiangya Hospital, Changsha, 410008 Hunan China

**Keywords:** Drug safety, Osteoporosis, Metabolic bone disease

## Abstract

Atypical femur fracture (AFF) is a rare but catastrophic adverse event first reported in the long-term use of alendronate, one of the most commonly used drugs for osteoporosis currently. However, further evidence is needed to learn more regarding other common anti-osteoporosis drugs and the risk for AFF. In this study, reports of AFF were identified from Food and Drug Administration Adverse Event Reporting System database. Disproportionality analyses were performed to examine the reporting odds ratio (ROR), information component (IC) and adjusted ROR (adj. ROR) signals for AFF for common anti-osteoporosis drugs. A total of 1692 unique AFF reports were identified. The disproportionality signals (the lower bound of 95% confidence interval > 1 for ROR and adjusted ROR, and > 0 for IC) were detected for alendronate, denosumab, pamidronate, risedronate, zoledronate, ibandronate, and teriparatide while no signal was detected for raloxifene, abaloparatide, and romosozumab. When restricted in patients with osteoporosis, the disproportionality signals were still detected for alendronate, pamidronate, risedronate, denosumab, and ibandronate. Our results suggest that alendronate has the largest risk signal, while the risks varied among different bisphosphonates. In addition, denosumab was found statistically associated with AFF in both the entire database and patients with osteoporosis.

## Introduction

Osteoporosis is a common geriatric disease diagnosed by bone density decreasing and deterioration of bone microarchitecture^1,2^. Patients with osteoporosis are at a higher risk of suffering fragility fractures, which may lead to disabilities that severely impairing life quality and even increase mortality rate^3^. Therefore, treatments for osteoporosis and prevention of fractures are of great both medical and social importance, especially when the global population is aging rapidly with ever-increasing life expectancy.

Bisphosphonates, by inhibiting bone resorption, are the mainstream anti-osteoporosis therapies and have been proven to increase bone density and reduce vertebral and non-vertebral fractures effectively^4^. However, since 2005, atypical femur fracture (AFF), an unusual fragility fracture in the subtrochanteric region and femur diaphysis, has emerged as a rare but serious adverse event of bisphosphonate therapy^5,6^. Although the absolute risk of AFF is very low (ranging from 50 to 130 cases per 100,000 patient-years comparing to the common osteoporotic femoral fracture)^7^, surgical interventions are more frequently required^8^. And the public concern of AFF has subsequently led to the reduced use of bisphosphonate^9,10^. As the American Society for Bone and Mineral Research (ASBMR) established task force to develop the diagnostic criteria of AFF^7,11^, more evidence suggests an increased risk of AFF with long-term bisphosphonate use, mainly alendronate^12–14^. Aside from bisphosphonates, several other AFF cases were reported in patients receiving common anti-osteoporosis therapies, including denosumab^15,16^, odanacatib^17^, and romosozumab^18,19^. However, uncertainty remains regarding the association between AFF and common anti-osteoporosis drugs.

The Food and Drug Administration Adverse Event Reporting System (FAERS) is designated by FDA to aid in post-marketing safety surveillance of drugs and therapeutic biological products^20^. FAERS monitors adverse event reports submitted by healthcare professionals, consumers, and manufacturers, enabling early detection of rare, unexpected, and delayed adverse events that are difficult to identify in clinical trials^21–23^. To our knowledge, only a limited number of studies have attempted to review the AFF cases in FAERS^24,25^, and the association between AFF and various osteoporosis drugs were not fully assessed. In this study, we aim to describe the characteristics of AFF patients in FAERS and to investigate the possible link between AFF and common anti-osteoporosis drugs.

## Materials and methods

### Data source

This retrospective case/non-case pharmacovigilance study collects FAERS reports from January 2012 to March 2022. It is because the coding of adverse events was based on Medical Dictionary for Regulatory Activity (MedDRA) terminology, and the preferred term “Atypical femur fracture” was first included in 2011, no AFF report was found in FAERS until 2012.

**Figure 1:**
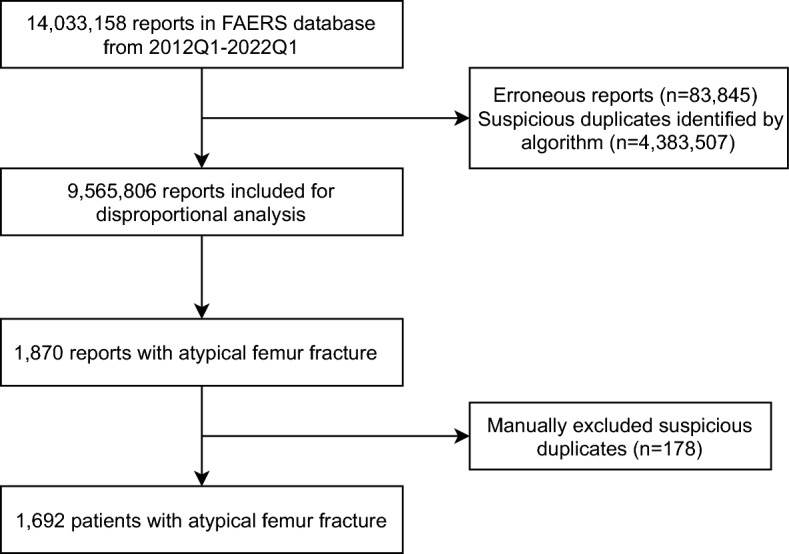
The flow chart of data retrieval and processing.

The duplicated reports in FAERS may undermine the reliability of disproportionality analysis^26^. We first removed specific reports indicated as deleted cases by FDA. An algorithm was applied to remove the suspected duplicated reports with the same drug-AE pairs based on gender, age, country, event date, and drug indications^27,28^. The cleaned database was further screened for the reports with “Atypical femur fracture” or “Atypical femur fracture bilateral” as the adverse event. For retrieved AFF reports, the details of drug use were further manually screened removed duplicated reports (e.g., using different brand names for the same drug). The flow chart of data processing is shown in Fig. 1.

Reports related to common anti-osteoporosis drugs, including each bisphosphonate, denosumab, raloxifene, teriparatide, abaloparatide, and romosozumab, were queried and retrieved. And the generic and brand names of drugs were mapped based on the Drugs@FDA database (https://www.fda.gov/drugs/drug-approvals-and-databases/about-drugsfda) and DrugBank Online Database (https://go.drugbank.com/).^29^ Some anti-osteoporosis drugs can also be used to treat SREs of cancers, mainly zoledronate and denosumab. For this reason, the indication information (reason for use) was also collected and categorized into osteoporosis, fracture, skeletal-related events (SREs), and others. We also extracted the time-to-onset (TTO) data of AFF with each anti-osteoporosis drug, which was defined as the time from the start date of drug use to the time of the event date. For reporting date given as year or year and month, inserting the first month or day as the missing data.

### Disproportionality and statistical analysis

Disproportionality analyses were conducted to assess whether there is an association between each anti-osteoporosis drug and AFF. If an association exists between a specific drug and an adverse event, it should have a higher frequency of reports than other drugs in the database, resulting in disproportionality. To further control for the confounder of osteoporosis states, we performed two separate analyses according to the indication of drugs. In the first scenario, we assess each anti-osteoporosis drug by comparing it with an aggregation of all other drugs in the FAERS database. In the second one, we compared each anti-osteoporosis drug with an aggregation of all other drugs used in patients with an osteoporosis indication without indications for fractures or SREs. In both scenarios, Reporting Odds Ratio (ROR), Information Component (IC), and adjusted ROR (adj. ROR) were used as measures of disproportionality. ROR is the pharmacovigilance equivalent to the odds ratio (OR) used in the case–control study. And IC is based on the non-frequentist Bayesian method that specifies the prior and posterior probabilities of suspect drugs and adverse events as new data are added to the database^30,31^. Previous studies have proven that ROR and IC are traditionally good indicators to assess the association between specific drugs and adverse events^32,33^. In addition, multivariable logistic regression model was used to calculate the adj. ROR. The models were adjusted for age, sex, reporting region, glucocorticoid use, and the use of each anti-osteoporosis drug. ROR and IC were computed based on a contingency table with raw data. However, nearly 35% of age data (3,301,711) and 10% of sex data (832,891) were missing, which may lead to serious bias if using imputed data. Thereby, the regression models were adjusted using complete case data of age and sex and removed cases with age < 0 or > 120 (erroneous or not representative). To further reduce confounders by race, we categorized the regions of case reporters into North America or Europe groups, Asia groups, and others. To confirm a disproportionality signal of a drug, the lower bound of 95% confidential interval should be > 1 for ROR and adj. ROR, and > 0 for IC based suggestions noted in similar studies^28,34^.

We further repeat our analyses in both scenarios using cases only reported by health professionals, excluding cases reported by consumers, lawyers, or others, as sensitivity analyses to assess the robustness of results.

The normality of variables was assessed using the Kolmogorov–Smirnov test. Continuous normally distributed variables were described as mean and standard deviation, while non-normally distributed variables were expressed as median and interquartile range (IQR). Categorical variables were compared using Chi-Squared test. All tests were two-sided and statistical significance was defined as P value < 0.05. Data processing was performed in SQL Server 2019, and statistical analysis was conducted using R software package version 4.0.

## Results

### Demographic of AFF patients

**Figure 2:**
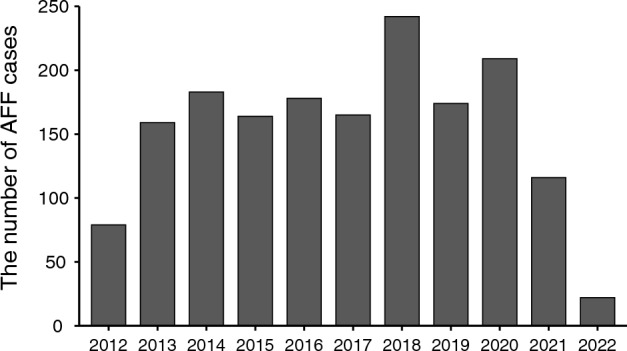
The number of AFF cases reported annually to the FAERS database from January 2012 to the March 2022.

The FAERS database contains 14,033,158 reports from the first quarter of 2012 to the first quarter of 2022. After removing the erroneous and duplicated reports, a total of 9,565,806 reports were retained. Among them, 1692 unique reports were finally identified, and the annual number of AFF reports is presented in Fig. 2.

The demographic characteristics of all 1692 AFF cases are shown in Table 1. Age information was provided for 1336 cases (Median age = 70, IQR: 62–78). 87.2% of AFF patients were female, and 6.1% were unknown. Most AFF cases occurred in the North Americas (n = 584, 34.5%), Europe (n = 532, 31.4%), and Asia (n = 493, 29.1%). And the majority of AFF cases were reported by health professionals (n = 1376, 81.3%), and fewer were reported by consumers or lawyers (n = 270, 16%). Among all AFF cases, 1668 (98.6%) had received anti-osteoporosis drugs. Furthermore, due to the longer treatment time for osteoporosis, patients may have used different anti-osteoporosis drugs. Nearly 3 of 4 AFF patients have used bisphosphonates (n = 1266, 74.8%), and alendronate was the most frequently reported drug in AFF patients, followed by denosumab and risedronate. 33.5% of AFF cases (n = 567) reported the use of denosumab, and 178 of them also reported any bisphosphonate use. We also identified 70 AFF patients on teriparatide, and only 10 of them did not report any use of bisphosphonate or denosumab.

**Table 1:** Characteristics of adverse event reports for atypical femur fracture in FAERS from January 2012 to March 2022.

Characteristic (n = 1692)	Value
Age, years (n = 1336)	
Median (IQR)	70 (62–78)
Gender	
Female	1475 (87.2)
Male	114 (6.7)
Unknown	103 (6.1)
Weight, Kg (n = 393)	
Median (IQR)	60 (53–69)
Reporting region	
North America	584 (34.5)
Europe	532 (31.4)
Asia	493 (29.1)
Oceania	62 (3.7)
South America	7 (0.4)
Others or missing	14 (0.8)
Reporter	
Health professional	1382 (81.7)
Lawyer/Consumer	270 (16.0)
Missing	40 (2.4)
Medications	
Any bisphosphonate	1266 (74.8)
Alendronate	808 (47.8)
Risedronate	329 (19.4)
Zoledronate	246(14.5)
Ibandronate	165 (9.8)
Pamidronate	89 (5.3)
Etidronate	20 (1.2)
Clodronate	0
Denosumab	567 (33.5)
Raloxifene	6 (0.4)
Teriparatide	70 (4.1)
Abaloparatide	1(0.1)
Romosozumab	3 (0.2)
Glucocorticoids	234 (13.8)

Values are No. (%) unless otherwise specified.

The indications of each anti-osteoporosis drug in AFF patients were varied (Supplementary Table S1). Overall, most AFF patients were used for preventing or treating osteoporosis (n = 1042, 61.6%), and a few with the indication of SREs (n = 194, 11.5%). For those treated for SREs, zoledronate (n = 118, 60.8%), pamidronate (n = 29, 14.9%), and denosumab (n = 105, 54.1%) were mainly used. Of note, the indication of teriparatide includes one AFF and two fracture nonunion cases.

**Figure 3:**
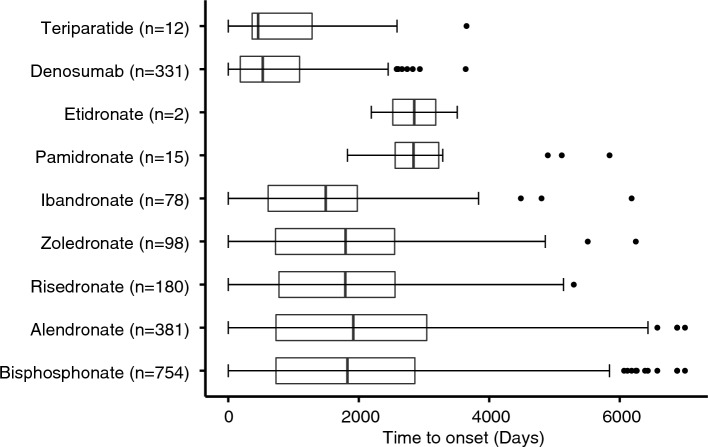
The time to onset of atypical femur fracture for each anti-osteoporosis drug.

A total of 1770 drug-AFF pairs for which the TTO could be calculated (Fig. 3), with a median time of 1277.5 days (IQR = 392.0–2365.8 days). Of note, the median TTO of alendronate (1916 days), risedronate (1793 days), and zoledronate (1797 days) were very close. Meanwhile, teriparatide (457 days) and denosumab (528 days) had a relatively shorter TTO.

### Disproportionality analysis

Disproportionality analyses were used to assess the association between each anti-osteoporosis drug and the reporting of AFF, and the characteristics of complete-case data were shown in Supplementary Table S2. In our first scenario, we compared each anti-osteoporosis drug against all the other drugs in the FAERS database. All anti-osteoporosis drugs as an aggregation had a ROR of 1990.73 (95% CI 1330.48–2978.62) and an IC of 4.85 (IC025 = 4.82). For each anti-osteoporosis drug, disproportionate signals (the lower bound of 95% confidential interval of ROR and adj. ROR > 1, and IC025 > 0) were detected for alendronate (adj. ROR = 57.49, 95% CI 51.35–64.33), risedronate (adj. ROR = 17.11, 95% CI 14.22–20.50), zoledronate (adj. ROR = 6.75, 95% CI 5.61–8.08), ibandronate (adj. ROR = 2.82, 95% CI 2.19–3.61), pamidronate (adj. ROR = 18.52, 95% CI 12.82–26.27), denosumab (adj. ROR = 24.66, 95% CI 21.64–28.07), and teriparatide (adj. ROR = 1.82, 95% CI 1.26–2.56). Etidronate and clodronate were the only two bisphosphonates not identified as statistically associated with the reporting of AFF compared to all other drugs in FAERS. Also, raloxifene, abaloparatide and romosozumab were not reported to be associated with the increased reporting of AFF in disproportionality analyses (Table 2).

**Table 2:** Disproportionality analysis of atypical femur fracture associated with anti-osteoporosis drugs in the entire FAERS database (scenario 1) and in patients with osteoporosis (scenario 2).

Medication	Scenario 1 (vs. all drugs in FAERS)			Scenario 2 (vs. drugs used in OP patients)		
	ROR (95% CI)	Adj. ROR (95% CI)	IC (IC025)	ROR (95% CI)	Adj. ROR (95% CI)	IC (IC025)
Alendronate	143.33 (130.25, 157.73)	55.37 (48.71, 62.90)	6.15 (6.07)	8.26 (7.30, 9.36)	10.94 (9.20, 13.02)	2.04 (1.95)
Risedronate	131.65 (116.62, 148.63)	17.11 (14.22, 20.50)	6.49 (6.33)	7.74 (6.62, 9.01)	6.14 (5.03, 7.46)	2.59 (2.4)
Zoledronate	27.56 (24.07, 31.56)	6.75 (5.61, 8.08)	4.5 (4.31)	0.82 (0.65, 1.03)	1.33 (1.00, 1.74)	−0.25 (−0.6)
Ibandronate	64.09 (54.54, 75.31)	2.82 (2.19, 3.61)	5.62 (5.37)	2.52 (2.08, 3.03)	1.72 (1.35, 2.18)	1.2 (0.94)
Pamidronate	103.82 (83.72, 128.75)	18.52 (12.82, 26.27)	5.98 (5.65)	13.31 (7.63, 21.63)	7.92 (3.42, 16.24)	3.17 (2.38)
Etidronate	335.48 (213.17, 527.97)	2.03 (0.90, 4.39)	5.18 (4.48)	18.15 (8.41, 34.63)	1.68 (0.65, 3.95)	3.16 (2.08)
Denosumab	51.23 (46.31, 56.69)	24.66 (21.64, 28.07)	5.05 (4.94)	1.26 (1.11, 1.44)	2.98 (2.45, 3.60)	0.24 (0.09)
Raloxifene	0.44 (0.20, 0.97)	0.11 (0.03, 0.30)	1.79 (0.44)	0.15 (0.04, 0.40)	0.22 (0.06, 0.59)	−2.5 (−4.49)
Teriparatide	6.33 (5.00, 8.04)	1.82 (1.26, 2.56)	2.56 (2.19)	0.10 (0.07, 0.13)	0.40 (0.27, 0.58)	−2.86 (−3.34)
Abaloparatide	0.66 (0.09, 4.72)	NA	−0.42 (−4.15)	NA	NA	NA
Romosozumab	5.29 (1.70, 16.42)	1.63 (0.40, 4.40)	1.71 (−0.28)	0.11 (0.02, 0.35)	0.25 (0.04, 0.78)	−2.83 (−5.34)

ROR = Reporting odds ratio; Adj. ROR = Adjusted ROR; IC = Information component; CI = Confidence interval; IC 025 = the lower bound of 95% CI of IC; OP = Osteoporosis; NA = Not applicable.

OP patients were selected based on the indication of drugs use while excluding patients with indications of fractures or cancers.

The Adj. ROR was adjusted with age, sex, reporting region, glucocorticoid use, and the use of each anti-osteoporosis drug.

The potential risk factors after adjustment were shown in Supplementary Table S3, and the adjusted OR were also significant for gender (OR for female vs. male, 5.78, 95% CI, 4.66–7.26), reporting region (OR for Asia vs. North America or Europe, 4.71, 95% CI 4.15–5.34 and others vs. North America or Europe, 1.98, 95% CI, 1.46–2.63). However, glucocorticoid use was not considered a risk factor for AFF in scenario 1 (OR = 0.80, 95% CI, 0.67–0.95).

In scenario 2, to control for effect caused by osteoporosis, disproportionality analyses were performed among anti-osteoporosis drugs compared with all other drugs used in osteoporosis patients. After excluding patients with indications of fractures or SREs, we identified 135,811 cases with osteoporosis indication, and 1021 (0.75%) of them suffered from AFF. In this analysis, only alendronate (adj. ROR = 10.94, 95% CI 9.20–13.02), risedronate (adj. ROR = 6.14, 95% CI 5.03–7.46), ibandronate (adj. ROR = 1.72, 95% CI 1.35–2.18), pamidronate (adj. ROR = 7.92, 95% CI 3.42–16.24), denosumab (adj. ROR = 2.98, 95% CI 2.45–3.60) still have a positive disproportionality signal for AFF. And zoledronate and teriparatide were no longer considered to be associated with the AFF in scenario 2. Also, the adjusted OR were significant for female (OR vs. male, 2.54, 95% CI, 1.85–3.60) and reporting in Asia (OR vs. North America or Europe, 2.42, 95% CI, 2.04–2.88). Consistent with scenario 1, glucocorticoid use was still not considered a risk factor (OR = 0.99, 95% CI 0.79–1.23).

Except for zoledronate in scenario 2 (adj. ROR = 1.72, 95% CI 1.27–2.30), the sensitivity analyses showed that using data only reported by health professionals did not change the results substantially (Supplementary Table S4).

## Discussion

In this study, we examined the entire collection of atypical femur fracture (AFF) reports in FAERS from January 2012 to March 2022 to investigate the potential association between common anti-osteoporosis drugs and atypical femur fracture using pharmacovigilance approaches. The results indicate that alendronate, risedronate, ibandronate, pamidronate, and denosumab were associated with an increased risk for AFF among osteoporosis patients. In addition to the aforementioned drugs, zoledronate and teriparatide were also noticed as relevant, if not directly associated, with AFF when compared with all other drugs in FAERS reports.

Atypical femur fracture has been considered as a rare and atypical adverse event of long-term bisphosphonate treatment for osteoporosis since 2005^5,10^. A recent cohort study included 196,129 postmenopausal women who received bisphosphonates (more than 90% were alendronate use) in Kaiser Permanente Southern California (KPSC) health care system, recorded that a total of 277 AFFs had occurred, and the risk for AFF increased with the longer duration of bisphosphonate treatment^12^. Concerns of AFF have also led to the suggestion proposing either stop bisphosphonates for a certain amount of time, called “drug holiday”, or switch to another anti-osteoporosis therapy^35^. The incidence of AFF among general population is very low, with a systemic review of 14 studies using ASBMR-defined criteria reporting an incidence rates of 3.0 to 9.8 per 100,000 person-years^36^. In FAERS, 1692 unique AFF cases were identified, accounting for approximately 0.02% of all patients with adverse events and 0.52% of reports associated with anti-osteoporosis drugs in the past decade.

Few studies separately analyzed the risk for AFF by different bisphosphonates other than alendronate. In practice, risedronate is one type of bisphosphonates that also commonly used orally and has been proven to reduce the risk of vertebral and non-vertebral fractures^22,37,38^. However, only few cases of risedronate-related AFF were reported^39–41^ and even no AFF case was recorded in a recent Cochrane review of 33 RCTs of risedronate^38^. Similarly, only a few reports set out to study the link between ibandronate^42^, pamidronate^43,44^, and etidronate^45^ with AFF. As an intravenous bisphosphonate, zoledronate can be prescribed to osteoporosis patients and cancer patients with SREs, with several studies discussing the intravenous bisphosphonate used in cancer patients and the risk of AFF^46–49^. In our study, zoledronate only contributes slightly in increasing the risk of AFF compared to drugs used in osteoporosis patients (adj. ROR = 1.33, 95% CI 1.00–1.74). However, nearly half of zoledronate-related AFF cases showed indications for SREs (n = 120), and this may contribute in part to the effect of zoledronate when compared with all other drugs in FAERS. Meanwhile, facing the enormous difficulty of precisely identifying SREs patients in the entire FAERS database, the specific risk of AFF induced by zoledronate and denosumab in patients with SREs is beyond the scope of this study.

As a monoclonal antibody targeting RANKL, denosumab is used to inhibiting RANK-RANKL interaction, reduce bone resorption, and preventing fracture^4,50–52^. In the FREEDOM Extension study, two denosumab-related AFF cases were adjudicated and generated an estimated incidence of 0.8 per 10,000 patient-years^16^. A few other literatures reported AFF related to denosumab^15,16,53^. The results of our study suggest a closer association between denosumab and AFF in both scenarios, and 70% of denosumab-associated cases were bisphosphonate naïve (n = 389). Anti-resorptive medication should be discontinued immediately after diagnosis of AFF^54^, but discontinuation of denosumab is associated with rebound bone loss and the risk of vertebral fractures^55–57^. Furthermore, transitioning from denosumab to teriparatide can lead to a significant decrease in the bone mass of distal radius and hip^58^, making denosumab-related AFF even trickier to treat.

Our study also identified 70 teriparatide-associated AFF cases, and 10 of them were without combined use of bisphosphonate or denosumab. The disproportionality analyses suggested an increased risk of AFF associated with teriparatide when compared to all other drugs in FAERS (adj. ROR = 1.82), but the effect was disappeared when compared to drugs used in osteoporosis patients (Table 2). Teriparatide is an anabolic agent that activates osteoblasts and osteoclasts, promoting bone formation^59^. Although the evidence is limited, several studies have supported the beneficial effect of teriparatide in AFF patients to accelerate fracture healing^60–64^. To our knowledge, the association between teriparatide or abaloparatide and the occurrence of AFF have not been fully explored yet. This may be because the teriparatide is rarely used alone in osteoporosis patients, and its treatment duration is typically restricted to 24 months due to concern about osteosarcoma. Additionally, transitioning from teriparatide to anti-resorptive therapy is the common practice, which may also obscure the relationship between teriparatide and AFF. Furthermore, the current diagnosis of AFF is mainly based on evidence obtained from patients receiving long-term bisphosphonate therapy, and it may not be applicable to other anti-osteoporosis drugs. For example, in the phase 3 long-term odanacatib fracture trial, 10 patients treated with odnacatib were adjudicated with AFF based on ASBMR criteria, but their characteristics, including significant lower BMD, no prodromal symptoms, and nearly all AFF occurred after fall^17^, were different from those of patients treated with bisphosphonates.

In our study, we did not observe disproportional signals for raloxifene, abaloparatide, romosozumab. However, it is noteworthy that abaloparatide and romosozumab were approved by FDA in 2017 and 2019, respectively. In the FRAME study, one AFF event happened 3.5 months after the first does of romosozumab^18^. And in the ARCH study, 6 patients were adjudicated to have AFF in the romosozumab-to-alendronate group (switch to alendronate after 12 months of romosozumab treatment)^19^. Since the risk of AFF may be associated to the longer duration of anti-osteoporosis drugs use, and the median TTO of AFF is more than 3 years, it is possible that longer follow-up periods are needed to observe any potential signals. In addition to each anti-osteoporosis drug, we also evaluated several potential risk factors for AFF. Our results are in consistence with previous studies marking Asian ancestry and female as risk factors for AFF ^12,14,65^. Several studies suggest an association of glucocorticoid with AFF^12,39,66^, whereas others do not^67–70^. Although 13.8% of AFF cases in our study reported glucocorticoid use, the adj. ROR fail to meet the cut-off value to stand as a disproportionality signal. However, the effect may be diluted or obscured due to the large amount of all glucocorticoid-related adverse events in the FAERS database (more than 0.6 million).

Less than half of the AFF cases in FAERS provided the TTO data, and the median value read about 3.5 years (median 1277.5 days, IQR, 392.0–2365.8 days). According to the KPSC cohort, 85% of AFF patients were exposed to bisphosphonate for at least 3 years, nearly 35% of which were exposed for more than eight years, and the risk of AFF plummets after discontinuation of bisphosphonate^12^. In our study, the TTOs of AFF induced by different bisphosphonates were similar, most of which lasted between 3 to 8 years. However, the TTOs of AFF induced by denosumab and teriparatide was averaging 528 days and 457 days respectively, which were obviously shorter than those associated with the alendronate (1916 days). That is consistent with the shorter duration treatment in previous case reports of AFF under denosumab^71–74^. Also, nearly 30% of denosumab-associated AFF cases reported having used bisphosphonate. And transitioning from bisphosphonates to denosumab, a more potent anti-resorptive drug, may increase the risk of AFF^75,76^.

However, we acknowledge that our study has several important limitations that should be taken into account when interpreting the results. First, unlike observational cohort studies, the number of patients exposed to drugs but without any adverse event in the pharmacovigilance database was limited^77^, resulting in potential incompleteness or inadequacy in the information on the background population. As a result, we were unable to determine the absolute incidence of adverse events accurately^78^. Furthermore, disproportionality analysis does not allow for causality assessment or accurately quantify the true risk of adverse events. Additionally, our results may have been affected by overreporting bias since we are not able to reassess the radiographic images according to ASBMR criteria. The lack of complete clinical information further restricted our ability to fully assess the patients’ characteristics and to adjust for potential confounders. To address these limitations, we conducted a dual-scenario analysis based on the full database and specifically focused on osteoporosis patients. We used adj. ROR to adjust for several confounders and performed a sensitivity analysis by excluding cases not reported by the health professionals to verify the robustness of our findings. Unfortunately, due to the lack of detailed data for the start and the cessation times of anti-osteoporotic drugs in a large subset of reports, we could not thoroughly analyze the effect of the exposure time of drugs and discontinuation. Instead, the TTO data for each anti-osteoporosis drugs were provided. Another inherent limitation of spontaneous databases is notoriety bias, as media coverage may increase the chance of physicians and consumers reporting^79^. However, it is noteworthy that we did not observe a significant uprise trend of AFF reports in the past decade. Despite these limitations, our study identified signals between common anti-osteoporosis drugs and AFF in one of the largest pharmacovigilance databases, which may provide clues and guidance for future studies of the safety and management of anti-osteoporosis drugs.

## Conclusion

In our study, we examined the disproportionality signals of common anti-osteoporosis drugs for AFF in FAERS database. Alendronate has the largest risk signals while risks varied among different bisphosphonates. The disproportionality signals of denosumab were detected in both entire database and patients with osteoporosis. Our results did not suggest the association between raloxifene, abaloparatide, and romosozumab with AFF. Although pharmacovigilance study is not able to establish causality, clinicians should be alert to the risk of atypical femur fractures in osteoporosis treatment, especially when the exposure are long-term use.

## Supplementary Information

Supplementary Information.

## Data Availability

The data that support the findings of this study are publicly available in the FDA Adverse Event Reporting System (FAERS) Quarterly Data Files, at https://fis.fda.gov/extensions/FPD-QDE-FAERS/FPD-QDE-FAERS.html.
