# Supplementary material for: Atypical femur fracture associated with common anti-osteoporosis drugs in FDA adverse event reporting system
Source: Sci Rep. 2023 Jul 5;13:10892. doi: 10.1038/s41598-023-37944-x (PMC10322871; doi:10.1038/s41598-023-37944-x)
Supplement: Supplementary file 1 — Supplementary Information. [file 41598_2023_37944_MOESM1_ESM.pdf]

Supplementary Material

Atypical Femur Fracture Associated with Common Anti-osteoporosis Drugs: A Pharmacovigilance Analysis of FAERS Database

Yao Xiao, Yiqian Chen, Yan Huang, Yuan Xiao *

*** Correspondence:** Yuan Xiao: xiaoyuan2021@csu.edu.cn

Content:

Table S1. Indication of anti-osteoporosis drugs used in patients reported atypical femur fracture.

Table S2. Characteristics of patients included in multivariate regression models for AFF (complete cases).

Table S3. Results of multivariable-adjusted models for atypical femur fracture in entire FAERS database (scenario 1) and in patients with indication osteoporosis (scenario 2).

Table S4. Results of sensitivity analysis for AFF using data reported only by health professionals in entire FAERS database (scenario 1) and in patients with indication osteoporosis (scenario 2).

Table S1. Indication of anti-osteoporosis drugs used in patients reported atypical femur fracture.

	**Osteoporosis**	**Fracture**	**SREs**	**Unknown**	**Others**
Alendronate	543	27	6	197	35
Risedronate	199	10	0	102	18
Zoledronate	73	4	120	40	9
Ibandronate	119	2	3	34	7
Pamidronate	14	1	29	34	11
Etidronate	3	0	0	10	7
Denosumab	321	12	112	108	14
Teriparatide	26	12	0	31	1
Abaloparatide	0	0	0	1	0
Romosozumab	2	1	0	0	0
Raloxifene	3	0	0	3	0

SREs = Skeletal-related events. Osteopenia, bone density decreased, low bone density, and osteoporosis prophylaxis were also included in the column of osteoporosis.

Table S2. Characteristics of patients included in multivariate regression models for AFF (complete cases).

	**Scenario 1 (All FAERS database)**			**Scenario 2 (with indication of OP)**		
	**Non cases**	**AFF cases**	**P-value**	**Non cases**	**AFF cases**	**P-value**
**Number**	6168370	1325		91593	808	
**Age**			<0.001			<0.001
≤45	1655384 (26.8)	63 (4.8)		2244 (2.4)	32 (4.0)	
46-55	941393 (15.3)	95 (7.2)		5567 (6.1)	37 (4.6)	
56-65	1316513 (21.3)	261 (19.7)		20104 (21.9)	141 (17.5)	
66-75	1232657 (20.0)	440 (33.2)		28870 (31.5)	273 (33.8)	
76-85	769848 (12.5)	364 (27.5)		25153 (27.5)	247 (30.6)	
>85	252575 (4.1)	102 (7.7)		9655 (10.5)	78 (9.7)	
**Reporing region**			<0.001			<0.001
North America or Europe	5234244 (84.9)	830 (62.6)		76507 (83.5)	562 (69.6)	
Asia	732284 (11.9)	436 (32.9)		11787 (12.9)	216 (26.7)	
Others	201842 (3.3)	59 (4.5)		3299 (3.6)	30 (3.7)	
**Gender**			<0.001			<0.001
Male	2467461 (40.0)	91 (6.9)		8431 (9.2)	39 (4.8)	
Female	3700909 (60.0)	1234 (93.1)		83162 (90.8)	769 (95.2)	
**Medication**						
Alendronate	38843 (0.6)	622 (46.9)	<0.001	10115 (11.0)	432 (53.5)	<0.001
Risedronate	12392 (0.2)	252 (19.0)	<0.001	3083 (3.4)	163 (20.2)	<0.001
Zoledronate	39435 (0.6)	208 (15.7)	<0.001	8492 (9.3)	65 (8.0)	0.256
Ibandronate	9915 (0.2)	127 (9.6)	<0.001	4876 (5.3)	100 (12.4)	<0.001
Pamidronate	3794 (0.1)	55 (4.2)	<0.001	133 (0.1)	9 (1.1)	<0.001
Etidronate	248 (0.0)	18 (1.4)	<0.001	44 (0.0)	8 (1.0)	<0.001

Denosumab	69006 (1.1)	460 (34.7)	<0.001	29494 (32.2)	283 (35.0)	0.095
Raloxifene	4817 (0.1)	4 (0.3)	0.015	1583 (1.7)	3 (0.4)	0.005
Teriparatide	36703 (0.6)	51 (3.8)	<0.001	24654 (26.9)	32 (4.0)	<0.001
Abaloparatide	4590 (0.1)	0 (0.0)	0.625	4164 (4.5)	0 (0.0)	<0.001
Romosozumab	1817 (0.0)	3 (0.2)	0.001	1460 (1.6)	2 (0.2)	0.004
Glucocorticoid use	467207 (7.6)	197 (14.9)	<0.001	6770 (7.4)	105 (13.0)	<0.001

Values are No. (%)

Table S3. Results of multivariable adjusted models for atypical femur fracture in entire FAERS database (scenario 1) and in patients with indication osteoporosis (scenario 2).

	Scenario 1		Scenario 2	
**Risk factors**	**Odds Ratio (95%CI)**	**P-value**	**Odds Ratio (95%CI)**	**P-value**
**Age (yr)**				
≤45	Reference		Reference	
46-55	1.80 (1.30, 2.51)	<0.001	0.43 (0.26, 0.71)	0.001
56-65	2.53 (1.92, 3.39)	<0.001	0.51 (0.34, 0.77)	0.001
66-75	3.84 (2.95, 5.09)	<0.001	0.69 (0.47, 1.03)	0.061
76-85	4.12 (3.15, 5.48)	<0.001	0.74 (0.51, 1.12)	0.136
>85	3.17 (2.28, 4.43)	<0.001	0.60 (0.39, 0.94)	0.022
**Gender**				
Male	Reference		Reference	
Female	5.78 (4.66, 7.26)	<0.001	2.54 (1.85, 3.60)	<0.001
**Reporting region**				
North America or Europe	Reference		Reference	
Asia	4.71 (4.15, 5.34)	<0.001	2.42 (2.04, 2.88)	<0.001
Others	1.98 (1.46, 2.63)	<0.001	1.20 (0.80, 1.73)	0.35
**Glucocorticoid use**				
No	Reference		Reference	
Yes	0.80 (0.67, 0.95)	0.012	0.99 (0.79, 1.23)	0.906

Table S4. Results of sensitivity analysis for AFF using data reported only by health professionals in entire FAERS database (scenario 1) and in patients with indication osteoporosis (scenario 2).

Risk factors	Scenario 1<br>Adj. OR (95% CI)	Scenario 2<br>Adj. OR (95% CI)
**Age (yr)**		
<45	Reference	Reference
45-55	1.68 (1.19, 2.39)	0.36 (0.21, 0.63)
55-65	2.34 (1.75, 3.19)	0.48 (0.31, 0.75)
65-75	3.55 (2.69, 4.77)	0.66 (0.44, 1.02)
75-85	3.45 (2.59, 4.66)	0.66 (0.44, 1.02)
>85	2.44 (1.71, 3.50)	0.51 (0.33, 0.83)
**Reporting region**		
North America or Europe	Reference	Reference
Asia	4.47 (3.90, 5.13)	2.24 (1.85, 2.70)
Others	2.50 (1.81, 3.38)	1.47 (0.97, 2.15)
**Gender**		
Male	Reference	Reference
Female	5.98 (4.77, 7.60)	2.67 (1.89, 3.89)
**Medication**		
Alendronate	44.77 (38.86, 51.53)	8.54 (7.09, 10.29)
Risedronate	21.02 (17.28, 25.45)	6.12 (4.93, 7.57)
Zoledronate	7.48 (6.16, 9.04)	1.72 (1.27, 2.30)
Ibandronate	4.31 (3.23, 5.68)	2.46 (1.88, 3.17)
Pamidronate	19.23 (13.20, 27.45)	6.72 (2.84, 14.14)
Etidronate	2.18 (0.91, 5.10)	1.61 (0.57, 4.13)
Denosumab	20.33 (17.65, 23.40)	2.33 (1.89, 2.86)
Raloxifene	0.07 (0.01, 0.28)	0.10 (0.01, 0.44)
Teriparatide	1.89 (1.17, 2.94)	0.61 (0.37, 0.95)
Abaloparatide	NA	NA
Romosozumab	1.43 (0.35, 3.83)	0.18 (0.03, 0.57)
Glucocorticoid use	0.66 (0.54, 0.79)	0.80 (0.62, 1.03)

NA = Not applicable
